# Retrospective evaluation of sociodemographic and clinical characteristics of patients with schizophrenia receiving clozapine monotherapy and clozapine combined with different antipsychotics

**DOI:** 10.1192/j.eurpsy.2024.596

**Published:** 2024-08-27

**Authors:** E. Yıldız, S. N. İspir, H. R. Demirel, S. Odabaş Ünal, M. Aydın

**Affiliations:** Department of Psychiatry, Selçuk University, Faculty of Medicine, Konya, Türkiye

## Abstract

**Introduction:**

Schizophrenia is a chronic mental disorder and clozapine is an atypical antipsychotic that can be used in treatment-resistant schizophrenia patients. However, treatment-resistant schizophrenia may also include patients with an inadequate response to clozapine.

**Objectives:**

In our study, we retrospectively analysed the sociodemographic and clinical characteristics of patients receiving clozapine monotherapy and patients receiving clozapine in combination with different antipsychotics. In this way, we aimed to evaluate the factors that influence the response to clozapine.

**Methods:**

Clozapine monotherapy and clozapine in combination with different antipsychotics were identified by retrospective chart review of patients followed up at the Schizophrenia and Other Psychotic Disorders Outpatient Clinic, Department of Psychiatry, Faculty of Medicine, Selçuk University. Sociodemographic and clinical characteristics were recorded and subjected to statistical analysis. The study was approved by the Ethics Committee of Selçuk University.

**Results:**

Of the 143 patients whose data were analysed, 60 (42%) were female. The mean age of the patients was 40.2±12.0 years and the mean duration of training was 10.4±4.3 years. 62 patients (43.4%) used long-acting antipsychotics. 90 patients (62.9%) were using clozapine, 52 (36.4%) were using clozapine as monotherapy, 5 (3.5%) were using clozapine together with another oral antipsychotics drug, and 33 (23.1%) were using clozapine together with a long-acting antipsychotic. No statistically significant difference was found when comparing mean age, age at first antipsychotic initiation, age at clozapine initiation and mean clozapine dose between patients using clozapine monotherapy (n=52) and patients using different antipsychotics in combination with clozapine (n=38). When the two groups were compared, a significant difference was found in the mean number of antipsychotics used before starting clozapine and the mean number of hospitalisations, with a lower number in the monotherapy group (3.1±1.4 vs 4.1±2.0, p=0.01 and 2.8±2.2 vs 4.5±3.2, p=0.006, respectively).

**Conclusions:**

It is important to assess the concept of treatment resistance appropriately in the treatment of schizophrenia patients. The results of our study suggest that starting clozapine treatment promptly in treatment-resistant patients may increase the likelihood that patients will benefit from clozapine and reduce the need for additional treatments. Although our data and criteria for evaluating response to treatment are limited, it is important to draw attention to the clinical results of proceeding in accordance with the guidelines in the treatment of schizophrenia. Evaluating the response to clozapine treatment needs studies with stronger data and larger sample sizes.

**Disclosure of Interest:**

None Declared

